# Emotional Intelligence in Spanish Elite Athletes: Is There a Differential Factor between Sports?

**DOI:** 10.3390/sports11080160

**Published:** 2023-08-18

**Authors:** Daniel Mon-López, Cecilia Blanco-García, Jorge Acebes-Sánchez, Gabriel Rodríguez-Romo, Moisés Marquina, Adrián Martín-Castellanos, Alfonso de la Rubia, Carlos Cordente Martínez, Jesús Oliván Mallén, María Garrido-Muñoz

**Affiliations:** 1 Deporte y Entrenamiento Research Group, Departamento de Deportes, Facultad de Ciencias de la Actividad Física y del Deporte, Universidad Politécnica de Madrid, Calle Martín Fierro, 7, 28040 Madrid, Spain; daniel.mon@upm.es (D.M.-L.); cecilia.blanco@upm.es (C.B.-G.); gabriel.rodriguez@upm.es (G.R.-R.); moises.mnieto@upm.es (M.M.); adrimartincast@gmail.com (A.M.-C.); carlos.cordente@upm.es (C.C.M.); jesus.olivan@upm.es (J.O.M.); maria.garrido.munoz@upm.es (M.G.-M.); 2 Faculty of Health Sciences, Universidad Francisco de Vitoria (UFV), Pozuelo de Alarcón, 28223 Madrid, Spain; j.acebes.prof@ufv.es; 3 Department of Physical Activity and Sports Science, Alfonso X El Sabio University (UAX), 28691 Madrid, Spain

**Keywords:** psychological skills, performance, team sport, individual sport, biological sex

## Abstract

Emotional intelligence is a determinant factor in sports performance. The present study analysed differences in total emotional intelligence and its four dimensions in 2166 Spanish athletes (25.20 ± 10.17 years) from eight sports (volleyball, track and field, shooting, football, basketball, handball, gymnastics, and judo). A total of 1200 men and 966 women answered anonymously using a Google Forms questionnaire sent via WhatsApp about demographics and psychological variables. A Pearson correlation was conducted to assess the age–emotional intelligence relationship. An independent *T*-test and One-Way ANOVA were carried out to check for age differences between biological sex and sport and a One-Way ANCOVA to determine differences between sports controlled by age. Age differences were observed by sex and sport (*p* < 0.001). An association was found between age and emotional intelligence dimensions (*p* < 0.001), except for other’s emotional appraisal (*p* > 0.05). Judo was the sport with the highest levels of regulation of emotions, other’s emotional appraisal, use of emotion, and total emotional intelligence (*p* < 0.05). Generally, emotional intelligence was found to be more developed in individual sports than in team sports, except football. Consequently, psychological skills like emotional intelligence could be critical to achieving high performance, depending on the sport.

## 1. Introduction

Sport psychology focuses on the study of different variables that have an impact on an athlete’s performance, with the aim of improving it. Different studies have proposed that variables such as anxiety [1,2], stress [3,4], motivation [5,6], self-confidence [7] have a relationship with sporting performance. Thus, sport psychology will seek to understand how psychological factors affect sport performance and vice versa in order to develop strategies that can help improve these variables [8].

Although emotional intelligence has been a popular research topic, there is a wide diversity of numerous paradigms and consequent assessment tools [9,10,11,12,13]. However, there is some consensus on the definition of emotional intelligence, which is a person’s ability to manage emotions. This ability is studied in four dimensions: (i) self-emotional appraisal (SEA). Refers to an individual’s ability to understand his or her deep emotions and to be able to express them naturally; (ii) other’s emotional appraisal (OEA). Connected to an individual’s ability to perceive and understand the emotions of the people around him or her; (iii) use of emotion (UOE). Associated with an individual’s ability to make use of emotions by directing them towards constructive activities and personal performance; and (iv) regulation of emotions (ROE). Related to an individual’s ability to regulate emotions and control behaviour when experiencing extreme moods [11,14].

Even though it is acknowledged that women show higher values compared with men [15,16,17], some investigations show that these differences are not significant [18,19,20]. However, specifically in the field of physical activity and sport, several results point out the opposite, with women showing significantly lower data [21,22,23]. Anyway, Fernandez-Berrocal et al. [24] argued that when age is controlled for, these relationships tend to disappear. This is because there appears to be a significantly positive correlation between emotional intelligence and age [25]. Some comparative studies have shown that adults have better emotional regulation than young people, which allows them to develop better mental health [26,27].

In the sports field, Laborde et al. [28] described an emotion as an organised psychophysiological reaction that evaluates ongoing contextual relationships. Numerous authors have studied emotional intelligence in sport and concluded that it is a variable that can significantly influence sport performance [22,29,30,31,32,33]. Specifically, Laborde et al. [34] stated that the type of sport has different psychological requirements. Thus, in individual sports, what each individual does or decides will not be compensated by any partner. This means that the athlete will bear the full weight of his or her decisions. Instead, team sports can compensate for their psychological requirements. In this line, Crombie et al. [35] defended how team emotional intelligence (i.e., the sum of emotional intelligence) predicts sport performance. Some studies have looked at emotional intelligence differences between sports. In relation to whether the type of sport was individual or team sport, most studies found no significant differences [36,37,38,39]. However, Castro-Sánchez et al. [40] found that athletes in team sports with contact (e.g., handball, football, or basketball) showed higher levels of emotional management than those in individual sports (with or without contact). Szabo and Urban [41], in their study of combat sports, concluded that boxing and combat sports in general may foster EI development. This could be because certain task-oriented motivational climates positively influence levels of emotional intelligence and anxiety, especially in contact sports [42]. EI can be developed through sport, insofar as sport, in many of its stages, is framed in educational or training stages [43]. Furthermore, it is important to focus attention on the risks that athletes present in terms of IE and well-being, as optimal performance is associated with pleasant emotions and dysfunctional performance with unpleasant emotions [44].

Although these studies have sought to categorise sports, it is recognised that each sport is different. For example, a study of volleyball has shown that the higher the emotional intelligence, the better the performance of male and female players [45]. With regard to track and field, Lu et al. [46] correlated emotional intelligence with lower levels of pre-competitive anxiety. In relation to shooting and archery sports, Sudarshan and Nagre [47] conducted research comparing both sports and found that archers showed significantly higher emotional intelligence. However, it should be noted that the sample was very small. It is worth highlighting the work developed by Berastegui-Martínez and Lopez-Ubis [48] on intervention in professional female football players, achieving an improvement in emotional intelligence levels and subsequent performance. In relation to gymnastics, a study conducted by Tatsi et al. [49] with acrobatic gymnasts aged 9 to 18 years showed that these athletes presented high emotional intelligence levels despite their age. Similar results have also been found with athletes in extreme and high-intensity sports such as ultramarathons, climbers, and cyclists [50,51,52,53] or combat sports [22,23,29,41]. However, no research has been found that treats the characteristics of each sport as unique and compares levels of emotional intelligence.

Due to the limited number of studies, a meta-analysis by Kopp and Jekauc [54] was unable to obtain conclusive results in the comparison between sport types, highlighting the need for further research. Therefore, the aim of this research was to analyse the differences in total emotional intelligence and its four dimensions (SEA, OEA, UOE, and ROE) in a large sample of Spanish-federated athletes from eight different sports (volleyball, track and field, shooting, football, basketball, handball, gymnastics, and judo), controlling for sex and age.

## 2. Materials and Methods

### 2.1. Participants

A total of 2232 athletes participated in the study by completing the questionnaires. Athletes participated in eight sports disciplines: volleyball, track and field, Olympic shooting, football, basketball, handball, gymnastics, and judo. The participant’s inclusion criteria were that all athletes must have a license from their official Spanish Federation and have competed in the 2020 season. The exclusion criteria were the following: (a) records with missing data (*n* = 13); and (b) staff and coaches’ responses were excluded (*n* = 53). The final sample was 2166 athletes (age 25.20 ± 10.17 years), of whom 1200 were men (age 27.85 ± 11.10 years) and 966 were women (age 21.90 ± 7.68 years). Considering the data provided by Consejo Superior de Deportes—CSD [55], this study was carried out with a more equitable—balanced—sample in relation to the sex comparison (77%/23% federation men/women licences, respectively). In addition, athletes were divided according to their nationality (Spanish or not), their residence country, and whether they had been called up to represent their national team (Table 1). This study was approved by the ethics committee of the Universidad Politécnica de Madrid.

### 2.2. Instrument and Variables

A questionnaire was used to collect information from the eight sports cited above. The demographic and training questionnaire designs were carried out independently by two professional coaches with wide international experience. Later, both questionnaires were discussed by the two researchers, who developed an initial version of the combined questionnaire. This version was tested in a pilot study involving four athletes (two men and two women), and their feedback was used to revise and modify the survey by a third external sports expert. The definitive version was prepared by consensus among the three experts and consisted of 23 items structured as follows: Q1–Q7 were demographic questions adapted from [56,57] and were single-choice. Moreover, Q8–Q23 were Likert-type scales from 1 (totally disagree) to 7 (totally agree) that belong to the Wong Law Emotional Intelligence Scale Short Form (WLEIS-S), adapted and validated in Spanish by Extremera-Pacheco et al. [58]. Although the approximate time to complete the questionnaire was 5 min, unlimited time to fill out the survey was provided to the athletes.

The study variables were distributed into two areas: demographic and psychological. Demographic variables were sport (volleyball, track and field, olympic shooting, football, basketball, handball, gymnastics, or judo), age (years), sex (male or female), nationality (Spanish or foreign), residence country (Spain or other countries), play role (coach or athlete), and sport level (athlete called up by the national team in the last two years, yes or no). With regard to the psychological variables, emotional intelligence (EI) was analysed in five areas: own emotions—SEA (α = 0.855), evaluation of others’ emotions—OEA (α = 0.779), use of emotion—UOE (α = 0.852), regulation of emotions—ROE (α = 0.883), and total emotional intelligence—EI Total (α = 0.873).

### 2.3. Procedures

The snowball sampling technique was used to send the final version of the survey through a Google Forms questionnaire to the athletes and technical staff [59]. A follow-up was sent two days later with the aim of increasing the response rate [60]. All the survey invitations and follow-ups were sent via WhatsApp. The questionnaire was open for ten days (after which no surveys were accepted) and anonymous to verify the honesty of the answers. The minimum estimated response rate was 55.70%, with 210 surveys finally registered and a maximum hypothetical number of responses associated with the invitations sent out of 377. According to Deutskens et al. [60] and Mavletova and Couper [61], the estimated response rate could be considered adequate or good. However, since it is not possible to know the exact response rate, there are still convincing reasons to consider a very good data set of the actual responses from 175 Spanish players [62]. All participants signed an informed consent form before completing the survey.

### 2.4. Data Analysis

Variables were described using the mean and the standard deviation (*X ± SD*). The normal distribution of the variables was checked using the Kolmogorov–Smirnov test, and the homogeneity of variance was tested using Levene’s test. Values higher than three standard deviations were excluded to avoid extreme outliers. An independent *T*-test was carried out to check for age differences between sexes, while a One-Way ANOVA was used to compare the ages between sports. Furthermore, Pearson correlation coefficient was used to assess the linear relationship between age and EI dimensions. Lastly, a One–Way ANCOVA was conducted to determine significant differences between sports on the EI, controlling for age. The post-hoc analysis was conducted using the Bonferroni test. The effect size was estimated using Cohen’s d index (*d*) in the comparison of sexes, establishing two cut-off points: medium effect (0.30) and large effect (0.60). On the other hand, the effect sizes were determined using the Eta squared (*η*^2^) for sport groups based on the following criteria [63]: small effects (<0.06); moderate effects (0.06–0.14); and large effects (>0.14). The collected data were studied using the software Statistical Package for Social Science (SPSS, IBM Corporation; Armonk, NY, USA), 25.0. version. The level of significance was set at 0.05.

## 3. Results

Age was checked by sex and sport (Table 2). Differences were observed in age by sex, with men older than women (t_2164_ = 14.17; *p* < 0.001; *d* = 0.613). Moreover, age presented also significant differences by sport (*F*_7,2158_ = 186.38; *p* < 0.001; *η*^2^ = 0.377). Bonferroni post hoc analysis showed that gymnastics presented the lowest age, with significant differences from the rest of the groups (*p* < 0.001, in all comparisons). In contrast, shooting and judo were the oldest ones in all comparisons (*p* < 0.001, both). In addition, handball players showed differences with football (*p* < 0.01), with higher values for footballers; volleyball and track and field also presented differences with football (*p* < 0.001, both) and basketball (*p* < 0.01, both), being younger than them.

Additionally, a correlation was found between EI and players’ age (Table 3). A positive association was found in men between age and IE dimensions, such as SEA, UOE, ROE, and EI Total, with values ranging from (*r* = 0.159 to 0.218, all *p* < 0.001). Similarly, SEA, UOE, ROE, and EI Total presented a positive association in women, with values ranging from (*r* = 0.192 to 0.272, all *p* < 0.001). However, OEA did not show any relationship with age, regardless of sex (*p* > 0.05).

Table 4 presents the relationship between age and EI, controlling for age as a covariable by sex and for the general population. Moreover, age’s effect analysis was also included to confirm the relation between age and EI.

### 3.1. General Analysis

Age was significantly related to SEA (*F*_7,2157_ = 67.03, *p* < 0.001; *η*^2^ = 0.030; *β* = 0.022), UOE (*F*_7,2157_ = 41.75, *p* < 0.001; *η*^2^ = 0.015; *β* = 0.017), ROE (*F*_7,2157_ = 54,87, *p* < 0.001; *η*^2^ = 0.025; *β* = 0.024), and EI Total (*F*_1,2155_ = 55.84, *p* < 0.001; *η*^2^ = 0.025; *β* = 0.015), although it was not related to OEA (*p* > 0.05). Moreover, there was a significant effect of sports when the age was controlled on SEA (*F*_7,2157_ = 6.41, *p* < 0.001; *η*^2^ = 0.020), UOE (*F*_7,2157_ = 14.85, *p* < 0.001; *η*^2^ = 0.036), ROE (*F*_7,2157_ = 7.33, *p* < 0.001; *η*^2^ = 0.023), and IE Total (*F*_7,2155_ = 10.52, *p* < 0.001; *η*^2^ = 0.033. However, although age was not significantly related to OEA, sport presented significant differences (*F*_1,2153_ = 56.35, *p* < 0.001; *η*^2^ = 0.020) (Table 4).

**Figure 1:**
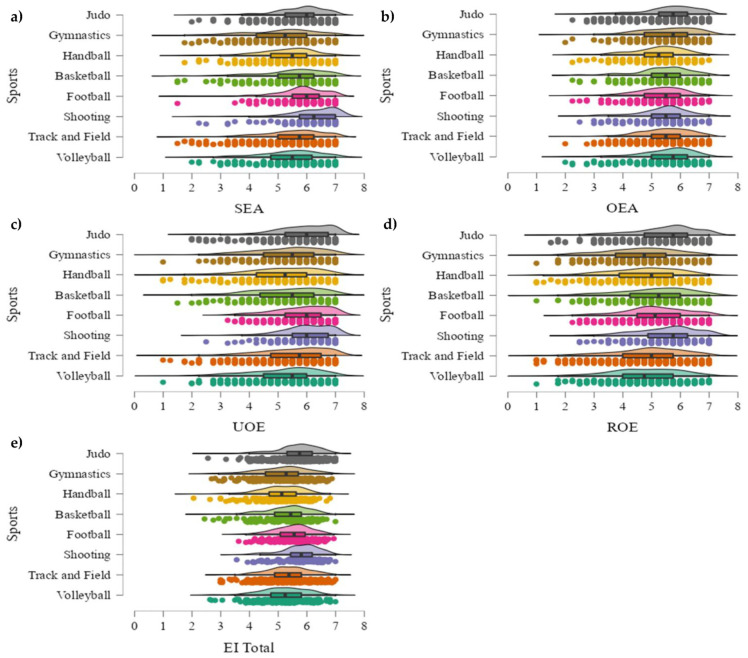
(**a**–**e**) Graphical representation of emotional intelligence markers in general population by sport. (**a**) = responses by sport in self-emotional appraisal (SEA); (**b**) = responses by sport in other’s emotional appraisal (OEA); (**c**) = responses by sport in use of emotion (UOE); (**d**) = responses by sport in regulation of emotions (ROE); (**e**) = responses by sport in total emotional intelligence (EI Total).

Post hoc tests (Figure 1) showed, with regard to SEA, that football presented higher results than handball (*p* < 0.001; *d* = 0.55), gymnastics (*p* < 0.001, *d* = 0.53), volleyball (*p* < 0.01; *d* = 0.35), track and field (*p* < 0.05; *d* = 0.25), and basketball (*p* < 0.05, *d* = 0.33). Furthermore, gymnastics showed lower values than judo (*p* < 0.01; *d* = 0.34), shooting (*p* < 0.01; *d* = 0.43), and track and field (*p* < 0.05; *d* = 0.25). Additionally, handball got a worse score than judo (*p* < 0.01; *d* = 0.33) and shooting (*p* < 0.05; *d* = 0.45). On the other hand, judo showed higher values than football (*p* < 0.001; *d* = 0.40), handball (*p* < 0.001; *d* = 0.45), and basketball (*p* < 0.05; *d* = 0.28) in OEA comparisons. Furthermore, handball got a worse score than gymnastics (*p* < 0.01; *d* = 0.35), volleyball (*p* < 0.05; *d* = 0.30), and track and field (*p* < 0.05; *d* = 0.30). In relation to UOE analysis, judo obtained better values than volleyball (*p* < 0.001; *d* = 0.49), basketball (*p* < 0.001; *d* = 0.47), handball (*p* < 0.001; *d* = 0.57), and gymnastics (*p* < 0.05; *d* = 0.32). Handball also showed differences with football (*p* < 0.001; *d* = 0.60), track and field (*p* < 0.001; *d* = 0.33), and shooting (*p* < 0.05; *d* = 0.44), obtaining a worse score. Moreover, basketball got lower results than football (*p* < 0.001; *d* = 0.51) and track and field (*p* < 0.05; *d* = 0.23). In the same lane, volleyball presented worse values than football (*p* < 0.001; *d* = 0.52), track and field (*p* < 0.01; *d* = 0.26), and shooting (*p* < 0.01; *d* = 0.36). Similarly, gymnastics achieved a worse score than football (*p* < 0.05; *d* = 0.36). Considering ROE analysis, judo scored higher than volleyball (*p* < 0.001; *d* = 0.40), track and field (*p* < 0.001; *d* = 0.34), gymnastics (*p* < 0.001; *d* = 0.49), and handball (*p* < 0.001; *d* = 0.49). Furthermore, football presented higher values than handball (*p* < 0.05; *d* = 0.32), and gymnastics (*p* < 0.05; *d* = 0.33). Lastly, there was a significant difference for IE Total between judo and gymnastics (*p* < 0.001; *d* = 0.45), handball (*p* < 0.001; *d* = 0.64), basketball (*p* < 0.001; *d* = 0.40), track and field (*p* < 0.001; *d* = 0.29), and volleyball (*p* < 0.001; *d* = 0.44), scoring higher for judo. Furthermore, handball presented lower values than track and field (*p* < 0.001; *d* = 0.34), football (*p* < 0.001; *d* = 0.56), and shooting (*p* < 0.01; *d* = 0.47). Additionally, football scored higher than volleyball (*p* < 0.01; *d* = 0.34) and gymnastics (*p* < 0.05; *d* = 0.35).

### 3.2. Women’s Analysis

Age was related to SEA (*F*_1,957_ = 2.90, *p* = 0.005; *η*^2^ = 0.021; *β* = 0.034), UOE (*F*_1,950_ = 20.54, *p* < 0.001; *η*^2^ = 0.021; *β* = 0.026), ROE (*F*_1,957_ = 21.81, *p* < 0.001; *η*^2^ = 0.022; *β* = 0.028), and EI Total (*F*_1,957_ = 29.20, *p* < 0.001; *η*^2^ = 0.030; *β* = 0.020) in women. In contrast, age did not have a relationship with OEA (*p* > 0.05). Moreover, there was a significant effect of sports on SEA (*F*_7,957_ = 44.33, *p* < 0.001; *η*^2^ = 0.044), OEA (*F*_7,955_ = 2.92, *p* = 0.005; *η*^2^ = 0.021), UOE (*F*_7,950_ = 3.61, *p* < 0.001; *η*^2^ = 0.026), ROE (*F*_7,957_ = 2.43, *p* = 0.018; *η*^2^ = 0.017), and EI Total (*F*_7,957_ = 3.97, *p* < 0.001; *η*^2^ = 0.028) (Table 4).

**Figure 2:**
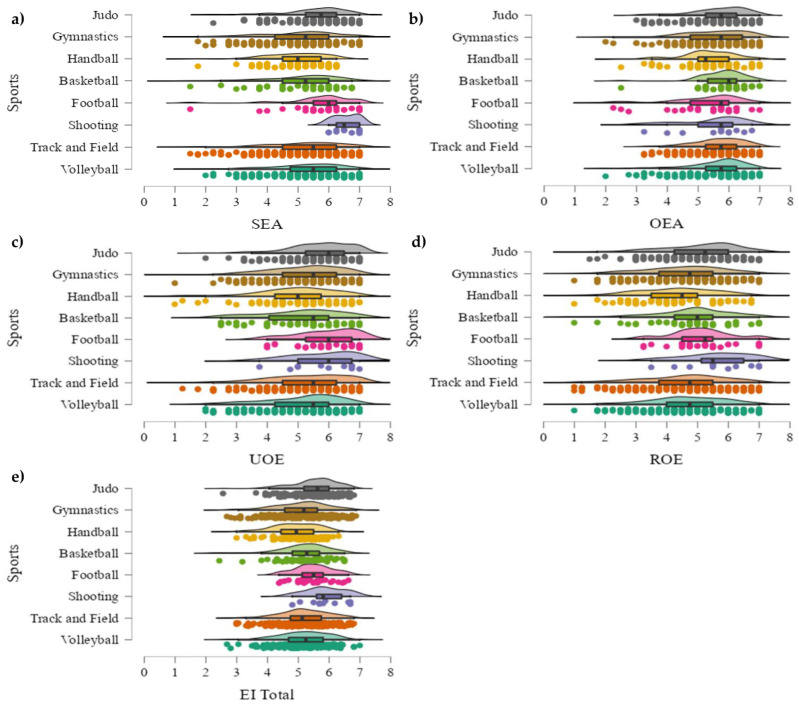
(**a**–**e**) Graphical representation of emotional intelligence markers in women by sport. (**a**) = responses by sport in self-emotional appraisal (SEA); (**b**) = responses by sport in other’s emotional appraisal (OEA); (**c**) = responses by sport in use of emotion (UOE); (**d**) = responses by sport in regulation of emotions (ROE); (**e**) = responses by sport in total emotional intelligence (EI Total).

Post hoc comparisons (Figure 2) pointed out that shooting presented higher SEA scores than all other sports: volleyball (*p* < 0.001; *d* = 0.57), track and field (*p* < 0.001; *d* = 0.56), football (*p* < 0.05; *d* = 0.31), basketball (*p* < 0.001; *d* = 0.81), handball (*p* < 0.001; *d* = 0.47), gymnastics (*p* < 0.001; *d* = 0.66), and judo (*p* < 0.001; *d* = 0.58). Also, judo had greater SEA values than gymnastics (*p* < 0.001; *d* = 0.23), handball (*p* < 0.001; *d* = 0.51), and track and field (*p* < 0.01; *d* = 0.12). Moreover, handball presented lower OEA values than track and field (*p* < 0.05; *d* = 0.44), basketball (*p* < 0.05; *d* = 0.64), and judo (*p* < 0.05; *d* = 0.45). With regard to UOE, judo presented higher values than volleyball (*p* < 0.05; *d* = 0.37) and handball (*p* < 0.01; *d* = 0.59), while football had greater scores than basketball (*p* < 0.05; *d* = 0.70). Furthermore, judo got a better value in ROE than handball (*p* < 0.05; *d* = 0.51), and handball presented a lower EI Total score than judo (*p* < 0.001; *d* = 0.69), football (*p* < 0.05; *d* = 0.77), and track and field (*p* < 0.001; *d* = 0.39).

### 3.3. Men’s Analysis

Age was also related to SEA (*F*_1,1191_ = 18.50, *p* < 0.001; *η*^2^ = 0.015; *β* = 0.013), UOE (*F*_1,1185_ = 6.70, *p* = 0.010; *η*^2^ = 0.006; *β* = 0.010), ROE (*F*_1,1191_ = 20.48, *p* < 0.001; *η*^2^ = 0.017; *β* = 0.017), and IE Total (*F*_1,1191_ = 19.17, *p* < 0.001; *η*^2^ = 0.016; *β* = 0.010) for men. Only OEA presented no relationship with men’s age (*p > 0.05*). Furthermore, there was a significant effect of sports on SEA (*F*_7,1191_ = 2.97, *p* = 0.004; *η*^2^ = 0.017), OEA (*F*_7,1189_ = 0.23, *p* < 0.001; *η*^2^ = 0.022), UOE (*F*_7,1185_ = 9.15, *p* < 0.001; *η*^2^ = 0.051), ROE (*F*_7,1191_ = 5.07, *p* < 0.001; *η*^2^ = 0.029), and IE Total (*F*_7,1191_ = 7.73, *p* < 0.001; *η*^2^ = 0.043) (Table 4).

**Figure 3:**
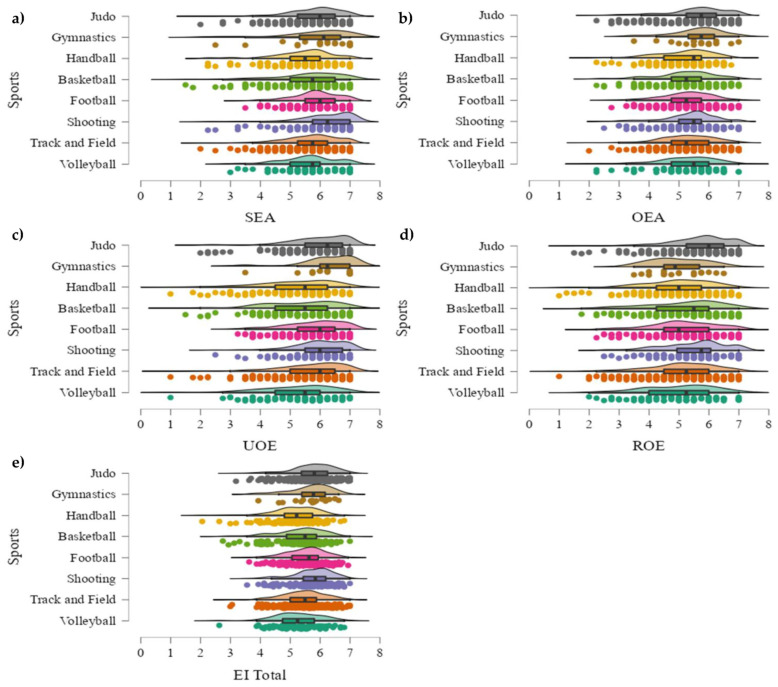
(**a**–**e**) Graphical representation of emotional intelligence markers in men by sport. (**a**) = responses by sport in self-emotional appraisal (SEA); (**b**) = responses by sport in other’s emotional appraisal (OEA); (**c**) = responses by sport in use of emotion (UOE); (**d**) = responses by sport in regulation of emotions (ROE); (**e**) = responses by sport in total emotional intelligence (EI Total).

Post hoc comparisons (Figure 3) pointed out that handball had worse SEA values than football (*p* < 0.01; *d* = 0.45) and shooting (*p* < 0.05; *d* = 0.45). However, judo showed higher OEA levels than basketball (*p* < 0.01; *d* = 0.39), handball (*p* < 0.01; *d* = 0.43), track and field (*p* < 0.05; *d* = 0.37), and football (*p* < 0.05; *d* = 0.35). Additionally, volleyball registered significantly worse UOE values than judo (*p* < 0.001; *d* = 0.58), gymnastics (*p* < 0.01; *d* = 0.92), track and field (*p* < 0.05; *d* = 0.40), shooting (*p* < 0.05; *d* = 0.52), and football (*p* < 0.01; *d* = 0.52). Basketball and handball also obtained lower values than track and field (*p* < 0.05; *d* = 0.35 and *p* < 0.001; *d* = 0.44, respectively), shooting (*p* < 0.05; *d* = 0.45/*p* < 0.01; *d* = 0.54), football (*p* < 0.01; *d* = 0.45/*p* < 0.001; *d* = 0.54), gymnastics (*p* < 0.01; *d* = 0.85/*p* < 0.001; *d* = 0.91), and judo (*p* < 0.001; *d* = 0.51/*p* < 0.01; *d* = 0.61). Nevertheless, judo obtained greater ROE values than handball (*p* < 0.001; *d* = 0.57), track and field (*p* < 0.001; *d* = 0.41), volleyball (*p* < 0.01; *d* = 0.51), football (*p* < 0.01; *d* = 0.40), shooting (*p* < 0.05; *d* = 0.39), and basketball (*p* < 0.05; *d* = 0.36). Associated with IE Total scores, judo presented higher values than volleyball (*p* < 0.001; *d* = 0.57), basketball (*p* < 0.001; *d* = 0.50), handball (*p* < 0.001; *d* = 0.69), and track and field (*p* < 0.05; *d* = 0.57). Moreover, handball had worse results than football (*p* < 0.01; *d* = 0.46), track and field (*p* < 0.01; *d* = 0.38), and shooting (*p* < 0.05; *d* = 0.48).

## 4. Discussion

The purpose of this study was to analyse the differences in total emotional intelligence and its four dimensions (SEA, OEA, UOE, and ROE) in a large sample of federated Spanish athletes in eight different sport modalities, controlling for sex and age.

In relation to women, shooting showed significantly higher values than the rest of the sports evaluated in the SEA variable—volleyball, track and field, football, basketball, handball, gymnastics, and judo. Although no preliminary studies comparing these sports have been found, Dal and Doğan [64] reported that shooters with higher emotional intelligence levels perceived projectile-induced physical and psychological stress as a challenge, leading to better performance. On the other hand, judokas also showed significantly higher values in SEA than track and field, handball, and gymnastics athletes. Likewise, judo showed significantly higher values than handball in OEA and ROE, and in total EI, handball showed higher values. Piskorska et al. [65] described combat sports as having differentiating characteristics from other sports that may be related to working on and improving emotional intelligence. Similar results were found by Reche-García et al. [66], who reported significantly higher emotional intelligence and resilience levels than individual sports (i.e., track and field and gymnastics) or team sports (i.e., handball). Furthermore, in relation to handball players, significantly lower values in total emotional intelligence and each of its dimensions were identified. The handball players also showed lower OEA values in relation to track and field basketball and judo and in total EI in relation to track and field and judo.

The results for men showed that judokas have the highest levels of emotional intelligence. Specifically, they seem to have a greater ability to perceive and understand the emotions of the people around them (OEA) than track and field, football, basketball, and handball. Also, they have a superior ability to make use of their emotions by directing them towards constructive activities and personal performance (UOE) compared with volleyball, basketball, and handball athletes. Likewise, judokas have a greater ability to regulate their emotions and control their behaviour when in extreme moods (ROE) compared with volleyball, shooting, track and field, football, basketball, and handball athletes. Moreover, they showed significantly higher EI Total values than volleyball, track and field, basketball, and handball. These results are in line with Mitic et al. [67], who affirm that judokas, compared with other athletes, had greater control over their emotions. According to these authors, this is due to the fact that the sport requires its competitors to have a high emotional charge but at the same time to control their emotions throughout the fight in order not to make possible mistakes. Furthermore, Stankovic et al. [68] showed in a study comparing team sports athletes and judokas that team sports athletes scored higher on emotionality and aggressiveness than judokas. That is, team athletes experience fear of physical danger, experience anxiety in response to life stresses, feel a need for emotional support from others, and feel empathy and sentimental bonds with others. Similarly, these results again support those of Reche-García et al. [66]. On the other hand, it seems that, in general, team sports (handball, basketball, and volleyball) show lower values compared with individual sports (gymnastics, shooting, or judo), with the exception of football. Nevertheless, scientific evidence did not find differences in emotional intelligence levels according to the type of sport [36,69]. In fact, Akelaitis and Malinauskas [70] stated in a study with 204 individual and 212 team athletes that team athletes showed higher self-awareness and self-regulation skills. It is worth noting that the sample was aged between 15 and 18 years, which could be relevant. However, in Acebes-Sánchez [71], with a similar sample in terms of number and nationality (1784 Spaniards of legal age who practised some type of sport), it was found that individual athletes showed significantly higher values than athletes in collective sports.

Controlling for comparisons by sex and age, the results presented judo as a sport with high OEA levels compared with collective sports [i.e., handball, basketball, and football]; ROE significantly higher than collective [i.e., volleyball and handball] and individual sports [i.e., track and field and gymnastics]; UOE higher than volleyball, basketball, handball, and gymnastics; and total EI compared with volleyball, basketball, handball, track and field, and gymnastics. Similar results were found previously by Acebes-Sánchez et al. [22], where judo athletes and high-performance judo athletes showed better EI than the rest of the studied groups (from different sports, active, and non-active individuals). According to Szabo and Urban [41], judo, as a combat sport, may foster emotional intelligence. It is worth noting that football, despite being a team sport, does not follow the same pattern. In fact, football players are higher in ROE than handball and gymnastics; in UOE than volleyball, basketball, handball, and gymnastics; and in total emotional intelligence than volleyball and handball. No similar results have been found previously. These results could be due to the fact that this sport enjoys greater resources (i.e., the presence of a sports psychologist).

The present study has several limitations. Firstly, the cross-sectional design meant that we were unable to infer causal relationships between the variables analysed; secondly, longitudinal studies would be necessary to establish cause-and-effect relationships and to study changes in EI during sports practise and in different competition contexts; and thirdly, differences with regard to control groups could not be analysed. On the other hand, EI has been assessed with a self-report tool, which means that it is treated as trait EI. However, this tool defines each dimension (SEA, OEA, UOE, and ROE) as ability EI. This should be taken into account when interpreting the results. It would also be interesting to carry out research with intervention to assess whether the mere practise of sport develops emotional competences or whether the specific development of these skills by professionals is necessary. Similarly, it would be interesting to carry out discriminant analyses to determine the general differences between these types of sports or factor analyses that could explain the variance between them.

This study presents the following strong points: On the one hand, the quantity and quality of the sample are high. No previous research is known to have made such a large comparison in terms of sample size and number of sports and athletes. On the other hand, this research highlights the realities of emotional intelligence according to different sports. From this reading, interventions can be developed in different sports to improve emotional intelligence levels. Also, it seems that some sports, such as judo, develop better emotional intelligence levels. This could be considered a way to promote this type of sport among young people, as it is a protective variable for mental health. As Gimeno et al. [58] highlighted, these results suggest the importance of psychological skills training to favour sports performance and injury prevention.

## 5. Conclusions

The main conclusion is that judokas have higher emotional intelligence levels compared with other sports, in both women and men. Also, when controlling for sex and age, these results remain the same. Although significant differences have been found between other sports, they have not been as noticeable or consistent as when comparing judo with other sports. However, it should be noted that female shooters show significantly higher SEA levels than the rest of the sports analysed.

## Figures and Tables

**Table 1:** Athletes sport characteristics (*n* and %) according to sex.

	Total		Men		Women	
	*n*	*%*	*n*	*%*	*n*	*%*
Sports Disciplines						
Volleyball	282	13.00	85	7.10	197	20.40
Track and Field	542	25.00	285	23.80	257	26.60
Olympic Shooting	143	6.60	132	11.00	11	1.10
Football	210	9.70	181	15.10	29	3.00
Basketball	183	8.40	133	11.10	50	5.20
Handball	215	9.90	138	11.50	77	8.00
Gymnastics	204	9.40	18	1.50	186	19.30
Judo	387	17.90	228	19.00	159	16.50
Nationality						
Spanish	2074	95.80	1154	96.20	920	95.20
Foreign	92	4.20	46	3.80	46	4.80
Residence country						
Spain	2136	98.60	1181	98.40	955	98.90
Others	30	1.40	19	1.60	11	1.10
National Selection						
Yes	339	15.70	138	11.50	201	20.80
No	1827	84.30	1062	88.50	765	79.20

**Table 2:** Age means, standard deviation, and significant differences by sex and sport.

		*n*	X	SD	Dif.
Sex					
Women		966	21.89	7.67	⚥ ***
Men		1200	27.85	11.10	
Sport					
Volleyball		282	21.85	7.49	C ***, D ***, E**, G ***, H ***
Track and Field		542	21.80	8.32	C ***, D ***, E**, G ***, H ***
Olympic Shooting		143	44.84	9.44	D ***, E ***, F ***, G ***, H ***
Football		210	25.75	5.21	F **, G ***, H ***
Basketball		183	24.64	6.27	G ***, H ***
Handball		215	22.82	5.96	G ***, H ***
Gymnastics		204	18.40	3.75	H ***
Judo		387	30.00	11.41	

Notes: Dif. = Differences between groups; ⚥ = differences between sexes; A = differences with volleyball; B = differences with track and field; C = differences with Olympic shooting; D = differences with football; E = differences with basketball; F = differences with handball; G = differences with gymnastics; H = differences with judo. * = *p* < 0.05; ** = *p* < 0.01; *** = *p* < 0.001.

**Table 3:** Correlation between age and total emotional intelligence (EI Total) and dimensions (SEA, OEA, UOE, and ROE).

	Age	SEA	OEA	UOE	ROE	EI Total
Age	—	0.272 ***	−0.032	0.192 ***	0.207 ***	0.237 ***
SEA	0.200 ***	—	0.201 ***	0.415 ***	0.538 ***	0.771 ***
OEA	0.053	0.244 ***	—	0.172 ***	0.173 ***	0.482 ***
UOE	0.159 ***	0.464 ***	0.271 ***	—	0.428 ***	0.728 ***
ROE	0.203 ***	0.441 ***	0.204 ***	0.397 ***	—	0.789 ***
EI Total	0.218 ***	0.745 ***	0.573 ***	0.766 ***	0.746 ***	—

Notes: SEA = self-emotional appraisal; OEA = other’s emotional appraisal; UOE = use of emotion; ROE = regulation of emotions; EI Total = total emotional intelligence. Men’s results are shown in the lower left corner, and women’s results are shown in the upper right corner * = *p* < 0.05, ** = *p* < 0.01, *** = *p* < 0.001.

**Table 4:** Differences in emotional intelligence dimensions (SEA, OEA, UOE, and ROE) and total (EI Total) according to sport and sex.

	SEA			OEA			UOE			ROE			EI Total		
	X	SD	Dif	X	SD	Dif	X	SD	Dif	X	SD	Dif	X	SD	Dif
Women															
Volleyball	5.31	1.10	C ***	5.63	0.91		5.15	1.23		4.68	1.23		5.19	0.79	
Track and Field	5.23	1.21	C ***, H **	5.68	0.81		5.27	1.29		4.56	1.37		5.19	0.78	
Shooting	6.57	0.40		5.48	1.10		5.82	1.06		5.64	1.13		5.88	0.63	
Football	5.70	1.16	C *	5.24	1.19		5.91	0.98	E *	5.12	0.99		5.50	0.60	
Basketball	5.16	1.15	C ***	5.83	0.73		5.07	1.30		4.83	1.29		5.20	0.80	
Handball	4.96	0.91	H ***, C ***	5.30	0.90	B *, E *, H *	4.94	1.31		4.33	1.26	H *	4.92	0.72	B *, D *, H ***
Gymnastics	5.05	1.19	H ***, C ***	5.57	1.00		5.22	1.16		4.56	1.28		5.10	0.87	
Judo	5.61	0.92	C ***	5.68	0.92		5.73	1.01	A *, F **	5.11	1.20		5.53	0.73	
Men															
Volleyball	5.61	0.92		5.23	0.99		5.25	1.19	B *, C *,D **, G **, H ***	4.99	1.20	H **	5.27	0.75	H ***
Track and Field	5.70	0.90		5.32	0.85	H *	5.70	1.09	E *, F ***	5.09	1.13	H ***	5.45	0.70	F **, H *
Shooting	6.16	0.98		5.37	0.88		6.00	0.86	E *, F **	5.49	1.08	H *	5.75	0.67	F *
Football	5.85	0.75		5.28	0.81	H *	5.81	0.94	E **, F ***	5.15	1.09	H **	5.52	0.63	F **
Basketball	5.58	1.18		5.21	1.00	H **	5.33	1.21	G **, H ***	5.16	1.27	H *	5.32	0.82	H ***
Handball	5.41	1.03	C *, D **	5.18	0.90	H **	5.20	1.24	G ***, H ***	4.91	1.21	H ***	5.18	0.77	H ***
Gymnastics	5.83	1.24		5.63	0.86		6.29	0.90		4.96	0.88		5.68	0.70	
Judo	5.81	0.87		5.61	1.00		5.95	1.00		5.69	1.06		5.77	0.71	
Total															
Volleyball	5.40	1.05	D **	5.51	0.95	F *	5.18	1.22	B **, C *, D ***, H ***	4.77	1.23	H ***	5.22	0.78	D **, H ***
Track and Field	5.48	1.08	D *, G *	5.49	0.85	F *	5.50	1.21	E *, F ***	4.84	1.27	H ***	5.33	0.75	F **, H **
Shooting	6.19	0.95	F *, G **	5.38	0.89		5.98	0.87	F *	5.51	1.08		5.76	0.67	F **
Football	5.83	0.82	E *, F ***, G ***	5.27	0.87	H ***	5.83	0.94	E ***, F ***, G *	5.15	1.08	F *, G *	5.52	0.62	F ***, G *
Basketball	5.47	1.18		5.38	0.97	H *	5.25	1.24	H ***	5.07	1.28		5.29	0.81	H ***
Handball	5.25	1.01	H **	5.22	0.90	G **, H ***	5.11	1.27	H ***	4.70	1.25	H ***	5.09	0.76	H ***
Gymnastics	5.12	1.21	H **	5.57	0.98		5.31	1.18	H *	4.59	1.25	H ***	5.15	0.87	H ***
Judo	5.73	0.90		5.64	0.97		5.86	1.01		5.45	1.15		5.67	0.72	

Notes: Dif = Differences between groups; A = differences with volleyball; B = differences with track and field; C = differences with shooting; D = differences with football; E = differences with basketball; F = differences with handball; G = differences with gymnastics; H = differences with judo; SEA = self-emotional appraisal; OEA = other’s emotional appraisal; UOE = use of emotion; ROE = regulation of emotions; EI Total = total emotional intelligence; * = *p* < 0.05, ** = *p* < 0.01, *** = *p* < 0.001.

## Data Availability

The participants in this study did not give written consent for their data to be shared publicly. Therefore, supporting data are not available due to the sensitive nature of the research.
